# PREDICTING DEMENTIA DIAGNOSIS FROM COGNITIVE FOOTPRINTS IN ELECTRONIC MEDICAL RECORDS USING MACHINE LEARNING

**DOI:** 10.1093/geroni/igad104.1294

**Published:** 2023-12-21

**Authors:** Jiayi Zhou, Hao Luo, Wenlong Liu, Huiquan Zhou

**Affiliations:** The University of Hong Kong, Hong Kong, Hong Kong; The University of Hong Kong, Hong Kong, Hong Kong; The University of Hong Kong, Hong Kong, Hong Kong; The University of Hong Kong, Hong Kong, Hong Kong

## Abstract

The cognitive footprint theory suggests that a person’s risk of dementia is affected by activities and events (footprints) across the lifespan. This study aimed to develop a clinical algorithm for predicting dementia diagnosis from cognitive footprints in electronic medical records (EMRs) using machine learning. Population-based EMRs from the Clinical Data Analysis and Reporting System in Hong Kong were employed. We included all patients with dementia diagnosed at 65+ between 2000 and 2018. Controls without dementia were matched (1:1) by age, sex, and index date. The prediction accuracy of a standard logistic regression model was compared with five machine learning models (LASSO, Random Forest, Multilayer perceptron, XGBoost, and LightGBM), using a wide range of established and exploratory risk factors identified at mid- ( < 65 years) and late-life (65+ years). A total of 261,116 individuals (59.3% female; mean age at index date [SD]: 82.70 [8.54]) with and without dementia were included. Compared with the logistic model (area under the curve [AUC]: 0.60), the predictive and diagnostic accuracy of dementia improved substantially in machine learning models. The Multilayer perceptron and LightGBM models showed comparable performance with the same test AUC of 0.720. LASSO (AUC: 0.765) and Random Forest (AUC: 0.768) showed a slightly worse performance. Antipsychotic drugs, education, antidepressants and head injury in late life were the top 4 important predictors in all models. The machine learning-based algorithm for predicting dementia can be used to identify patients with an increased likelihood of dementia to allow precise and timely primary interventions.

